# Supplementary material for: Asymptomatic bacteriuria and its associated fetomaternal outcomes among pregnant women delivering at Bugando Medical Centre in Mwanza, Tanzania
Source: PLoS One. 2024 Oct 3;19(10):e0303772. doi: 10.1371/journal.pone.0303772 (PMC11449372; doi:10.1371/journal.pone.0303772)
Supplement: S1 File — (PDF) [file pone.0303772.s001.pdf]

Procedures for Urine Culture

	Name:	Designation:	Signature:	Date:
**Written by:**				
**Reviewed by:**				
**Authorised by:**				

1.0 Purpose

This procedure provides instructions for performing culture, biochemical identification, and antimicrobial susceptibility testing at the Clinical Microbiology Laboratory at the Catholic University of Health and Allied Sciences in Mwanza, Tanzania.

2.0 Principle

Urine is the specimen most frequently submitted for culture in the laboratory. Commensals from the lower part of the urethra and the perineum can contaminate urine during sample collection. In female patients, the urine may also become contaminated with commensals from the vagina. Therefore, quantitative urine culture is mandatory.

Most urine specimens will contain fewer or more than 10^4 colony forming units per ml provided the sample has been collected with care to minimize contamination and examined soon after the collection before the commensals multiply significantly so that bacterial count will be unreliable.

3.0 Equipment

- Incubator
- Microscope

4.0 Reagents and Media

- Sheep Blood Agar
- Mac Conkey Agar

5.0 Materials

- 1 µl loop
- 10 µl loop
- Incubator
- Sterile wide mouth screw capped urine container

6.0 Safety Precautions

- All specimens must be regarded as potentially infectious. Use Good Laboratory Practice (GLP) when processing samples. Treat all samples as infectious and observe standard universal laboratory precautions.

7.0 Specimen

7.1 Specimen required.

- Clean catch midstream urine

7.2 Specimen collection

- Urine should be collected before antibiotic therapy.
- A first-morning sample is ideal; if this is not possible, the urine should be collected after being held for at least 3 hours.
- 5-10 ml urine should be collected as a **clean catch midstream** urine specimen.
- In a catheterised patient, the soft rubber connector between the catheter and the collecting tube is vigorously cleaned with 70% Methanol and urine aspirated by a sterile syringe. A specimen should not be obtained from the collecting bag.
- Clinicians for neonates and small children use suprapubic aspiration by disinfecting the skin over the bladder. Urine is aspirated by an 18-gauge short bevel spinal needle.

7.3 Specimen transportation and storage

- Specimen should be transported immediately to the laboratory after collection; if delay occurs for more than 2hours, the sample should be refrigerated at 2˚C to 8˚C

7.4 Specimen rejection

Do not process the following urine specimens:

- Delayed more than two hours without refrigeration or preservation.
- 24 hours’ urine collection
- Foley catheter tips, urine from the bag of catheterized patient
- Urine from bedpans
- Specimen received in non-sterile containers or leaking containers.
- Unlabeled and improperly labelled specimens
- Duplicate specimens collected on the same day.

8.0 Quality Control

- QC of media preparation

9.0 Procedure steps

- Use a sterile loop calibrated to deliver 0.001 ml.
- Mix urine well. Hold the loop vertically and immerse it just below the surface of the urine. Deliver the loopful onto the MCA and BA plates.
- Make a straight line down the centre of the plate and streak the urine by making a series of passes at 90 angles through the inoculum.

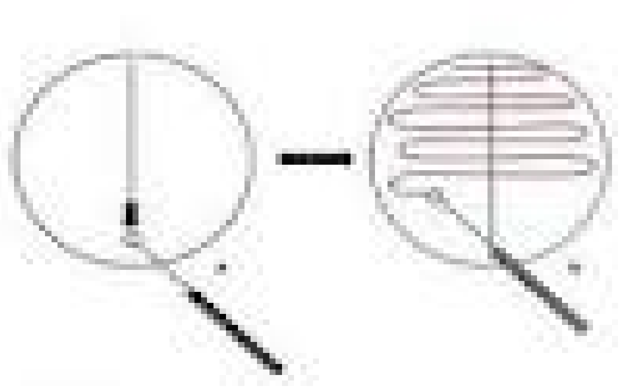

- Use a calibrated loop to deliver 0.001 ml for straight catheter, suprapubic, cystoscopy, and nephrostomy specimens.
- Incubate the agar plates overnight at 35° to 37°C for 18 -24 hours.
  - Perform appropriate tests for identification and antimicrobial susceptibility testing to significance positive, pure cultures. Do not identify resident urogenital flora to the genus or species level.

10.0 Interpretation and Reporting

The interpretation of bacterial growth in urine culture will depend on the type of urine sample being processed.
For the mid-stream urine and catheter urine:

Absolute number of bacteria colonies on the plate (using 1 µl) loop	Corresponding CFU/ml	Interpretation	Reporting
1 - 9	10^3	Indicates possible contamination	Report as "No significant bacteria growth."

10 - 99	10^4	Possible infection or contamination	Report the CFU/ml and proceed with identification and AST
≥ 100	10^5	Infection **(significant bacteriuria)**	Report the CFU/ml and proceed with identification and AST

- If there is growth of one or two co-dominant bacteria (i.e. each with $>10^4$ CFU/ml), proceed with identification and AST for each bacterium.
- If there are more than two bacteria types (i.e. ≥ 3 types), report as a "contaminated urine sample" and request another properly collected urine sample.
- Any growth from a suprapubic aspirated urine sample is significant, and the respective colonies must be subjected to identification and AST.
- It is important to interpret culture counts in relation to the patient's clinical condition.
- If there is no growth after 24 hours, report it as "No growth after 24 hours of incubation".

Note:
Appearance of urinary pathogens on MacConkey Agar:

- Lactose fermenters (pink colonies) or non-lactose fermenters (colourless).

  Appearance of urinary pathogens on CLED Agar:
  - *E. coli*: Yellow (lactose fermenter) opaque colonies, often with slightly deeper colored centre
  - *Klebsiella spp.*: large mucoid yellow (lactose fermenter) or yellow-white colonies
  - *Proteus*: translucent blue-grey colonies (non-lactose fermenter)
  - *Pseudomonas aeruginosa*: green colonies with rough periphery (characteristic colour)
  - *E. faecalis*: small yellow (lactose fermenter) colonies
  - *S. aureus*: deep yellow (lactose fermenter) colonies of uniform colour
  - *S. saprophyticus* and other coagulase-negative staphylococci: yellow to white colonies.

Note that Gram-positive bacteria do not grow on MacConkey with crystal violet agar but on either Blood Agar.

11.0 Possible pathogens in a urine sample

GRAM-POSITIVE BACTERIA

- *Staphylococcus aureus*
- *Staphylococcus saprophyticus*
- *Enterococcus* spp.
- *Haemolytic streptococci*

GRAM-NEGATIVE BACTERIA

- *Escherichia coli,*
- *Proteus* spp*,*
- *Klebsiella* spp. and other *Enterobacteriaceae*
- *Pseudomonas aeruginosa*

Note: *Neisseria gonorrhoea, Salmonella typhi and Salmonella paratyphi are not the primary causative agents of UTI but can be recovered as coincidental findings from urine culture,* which needs further investigation.

12.0 Antimicrobial susceptibility testing

Each identified bacterial species should be subjected to a conventional disk diffusion method based on the Clinical Laboratory Standard Institute (CLSI) version of 2020.

13.0 References

1. Murray PA, et al. Manual of Clinical Microbiology, 8th Edition, 2003, pp200-300,304-304
2. Mackie & McCartney, Practical Medical Microbiology, Churchill Livingstone, 14th Edition, 1996
3. Sherris John C., Editor, Medical Microbiology, An Introduction to Infectious Diseases, Elsevier, 1984, p606
4. Cheesbrough, M. District Laboratory Practice in Tropical Countries, 2nd Edition, Tropical Health Technology, 2006, p112-113
5. Clinical Microbiology Procedures Handbook. American Society for Microbiology. Washington D.C., USA, 3rd edition, 2010.
6. CLSI, Performance standards for antimicrobial susceptibility testing, 30th ed CLSI supplement M100 Clinical and Laboratory Standards Institute. 950 West Valley Road, Suit 2500, Wayne, Pennsylvania19087. USA. 2020.

APPENDIX 1: Gram-positive cocci aerobic identification flow chart

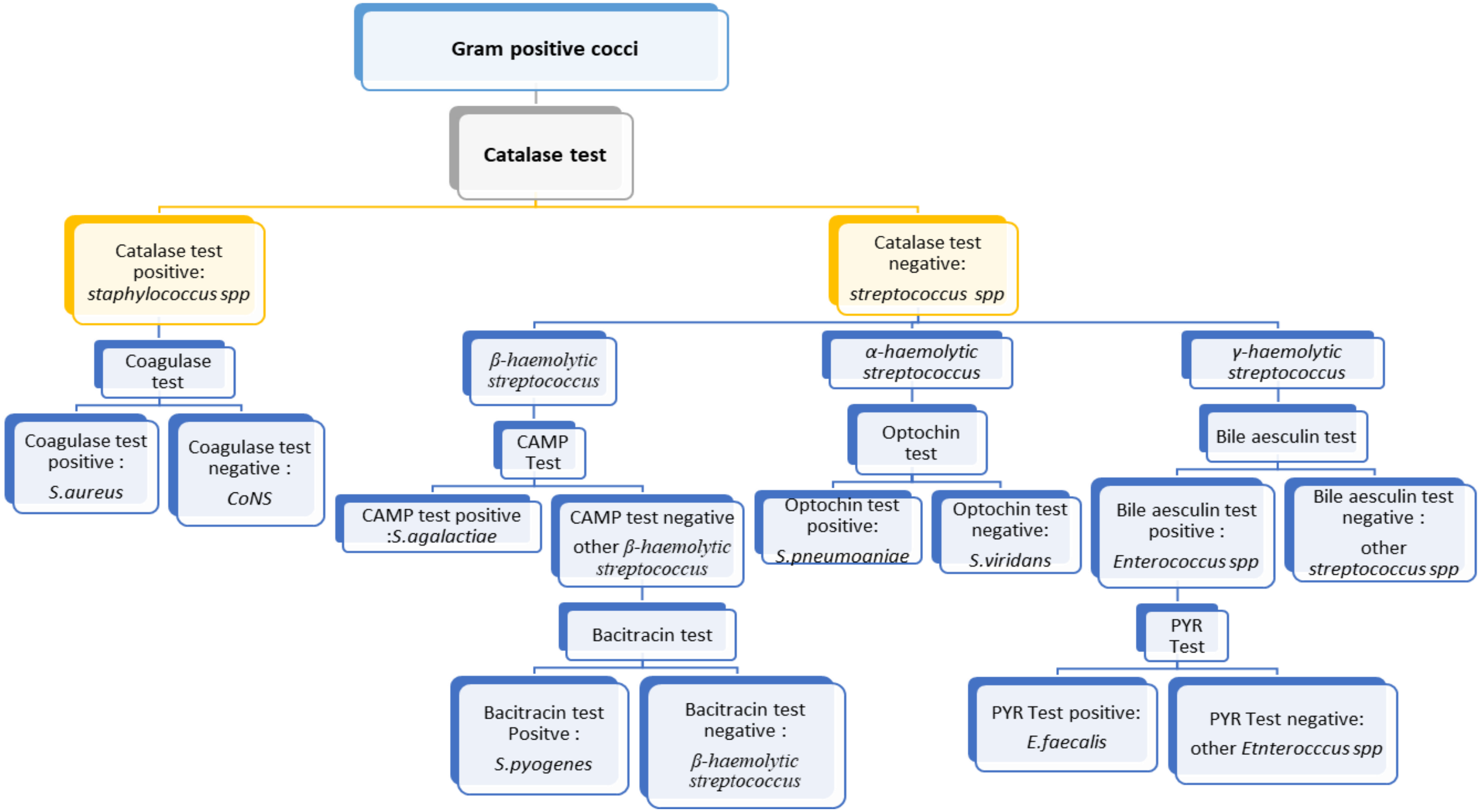

APPENDIX 2: Identification of gram-negative Enterobacteriaceae flow chart

Organism	MAC Reaction	TSI	Oxidase	H_2S	Gas	Motility	Indole	Urea	Citrate	Haemolysis	Comment

E.coli	LF	A/A	-	-	+	+	+	-	-	+/-	Often grows with precipitate bile salt on MCA
Enterobacter aerogenes	LF	A/A	-	-	+	+	-	-	+	-	Often resistant to Ampicillin and cephalosporin
Klebsiella pneumoniae	LF	A/A	-	-	+	-	-	+	+	-	Grow with very mucoid colonies
Klebsiella oxytoca	LF	A/A	-	-	+	-	+	+	+	-	
Citrobacter freundii	Late LF	A/A or K/A	-	+	+	+	-	+/-	+	-	

APPENDIX 3: Chart for Biochemical Identification of common Enterobacteriaceae and other enteric organism

Key:TSI-Triple Sugar Ion **,LF**-Lactose Fermenter ,**NLF**-Non-Lactose Fermenter,**A/A**-Acid/Acid,**K/A**-Alkaline/Acid,**K/NC**-Alkaline/No Change

Organism	MAC Reaction	TSI	Oxidase	H_2S	Gas	Motility	Indole	Urea	Citrate	Haemolysis	Comment
Serratia mercesens	**NLF**	**K/A or A/A**	**-**	**-**	**+**	**+**	**-**	**-**	**+**	**-**	**Red pigment at room temp on MHA**
Edwardsiella tarda	**LF**	**K/A**	**-**	**+**	**+**	**+**	**+**	**-**	**-**	**-**	
Proteus mirabilis	**NLF**	**K/A**	**-**	**+**	**+**	**+**	**-**	**+**	**+(weak)**	**-**	**Grow with swarming xters on BA**
Proteus vulgaris	**NLF**	**A/A or K/A**	**-**	**+**	**+**	**+**	**+**	**+**	**+/-**	**-**	**Grow with swarming xters on BA**
Salmonella sp	**NLF**	**K/A**	**-**	**+**	**+**	**+**	**-**	**-**	**+**	**-**	**Citrate pos (non-typhoid salmonella)**
Salmonella typhi	**NLF**	**K/A**	**-**	**Wk+**	**+**	**+**	**-**	**-**	**-**	**-**	
Shigella sonnei	**NLF**	**K/A**	**-**	**-**	**-**	**-**	**-**	**-**	**-**	**-**	
Other Shigella sp	**NLF**	**K/A**		**-**	**-**	**-**	**-**	**-**	**-**	**-**	
Vibrio cholerae	**NLF**	**A/A**	**-**	**+**	**+**	**-**	**-**	**-**	**-**	**+**	**String test-positive**
Vibrio parahaemolyticus	**NLF**	**K/A**	**+**	**-**	**-**	**+**	**+**	**-**	**+**	**+**	
P.aeruginosa	**NLF**	**K/NC**	**+**	**-**	**-**	**+**	**-**	**+**	**+**	**+/-**	**Green pigmentation on MHA**
Acinetobacter sp	**NLF**	**NC**	**-**	**-**	**-**	**-**	**-**	**-**	**-**	**-**	**Coccoide rods**
Morganella morganii	**NLF**	**K/A**	**-**	**-**	**+**	**+**	**+**	**+**	**-**	**-**	
Providencia spp	**NLF**		**-**	**-**		**+**	**+**		**+**	**-**	
Yersinia enterocolitica	**NLF**	**K/A**	**-**	**-**	**-**	**+(25°C) -(35°C)**	**+/-**	**+/-**	**-**	**-**	

MAC-Mac Conkey , **H_2S**-Hydrogen Sulphide

DOCUMENT CHANGE HISTORY

Proposed by	Section	Summary of Changes

I have read, understood and agree to follow the procedure as documented:

No	Name	Signature	Date

