# Supplementary material for: Asymptomatic bacteriuria and its associated fetomaternal outcomes among pregnant women delivering at Bugando Medical Centre in Mwanza, Tanzania
Source: PLoS One. 2024 Oct 3;19(10):e0303772. doi: 10.1371/journal.pone.0303772 (PMC11449372; doi:10.1371/journal.pone.0303772)
Supplement: Attachment — Submitted filename: Responses to Reviewers.docx [file pone.0303772.s003.docx]

**ADDRESSING REVIEWERS’ COMMENTS TO PLOS One**

| **S/N** | **REVIEWER/EDITORS COMMENTS** | **RESPONSES** | **ACTION** |
| --- | --- | --- | --- |
| **EDITORS COMMENTS** | | |  |
| 1. | We recommend that you deposit your laboratory protocols in protocols.io to enhance the reproducibility of your results | The protocol used for the analysis of urine samples from pregnant women is included as a supplemental file (Urine Culture Protocol: CUHAS Clinical Microbiology Laboratory). | Supplemental file 1: |
| 2. | Ensure that your manuscript meets PLOS One's style requirements, including those for file naming. The PLOS One style templates | This was adhered to as suggested by the editor and the PLOS One’s journal style. | Addressed throughout the revised manuscript. |
| 3. | Copyedit your manuscript for language usage, spelling, and grammar. | The grammar and language check has been performed by two different English speakers | Addressed throughout the revised manuscript. |
| 4. | Provide non-author contact information (phone/email/hyperlink) for a data access committee, ethics committee, or other institutional body to which data requests may be sent | Data from this study are attached in the support information section of this article. | Page 21  Supplementary file 2: |
| 5. | Review your reference list to ensure that it is complete and correct. If you have cited papers that have been retracted, please include the rationale for doing so in the manuscript text, or remove these references and replace them with relevant current references | References have been updated to the recent ones. None of the retracted papers in the reference list |  |
| **REVIEWER #1** | | |  |
| 1. | Background: Reference 1, indicates prevalence of UTI among pregnant women globally is old, update to recent data. | The reference has been changed to “Salari N, Khoshbakht Y, Hemmati M, Khodayari Y, Khaleghi A, Jafari F, et al. Global prevalence of urinary tract infection in pregnant mothers: a systematic review and meta-analysis. Public Health. 2023;224:58-65” | Page 2, line 44 & Reference #1 in pg 21 has been updated. |
| 2. | Methods: Why women with multiple pregnancies are excluded? And what does multiple pregnancies mean? Is it to show multigravidity or twin, or triple pregnancy at current pregnancy? | Multiple pregnancies were excluded to minimize confounders since multiple pregnancies are also known to cause prematurity and low birth weight. Multiple pregnancy means someone is carrying more than one fetus/baby at a time | No changes were made in the revised manuscript. |
| 3. | Age is categorized as < 30 and ≥ 30 but, your nation indicates ‘ranging from 15 to 45 years’. Brief or correct. | The country has indeed adopted the WHO reproductive age group ranging from 15-45 years and this was the age pattern in the data. However, dichotomization was done since the mean/SD age was 29±5.6. | No changes have been made to the revised manuscript |
| 4. | Table 1, variable parity categorized as Multipara and Primigravida: are contradicting, gravidity and parity are different. Please correct or brief. | This has been changed to gravidity as a variable categorized into primigravida and multigravida. | Table 1, Page 8. |
| 5. | In Tables 2, 3, and 4, what are the empty boxes and * indicates in the multivariable logistic regression analysis column? | The * in the empty boxes was put to denote that no multiple regression calculations were performed. To minimize confusion the * has been deleted. | * Not included in Tables 2, 3, and 4, page 9-12. |
| **REVIEWER #2** | | |  |
| 1. | References: the authors listed 39 references, 25 of which are over five years old. I suggest authors update their references with more recent studies that provide relevant information or findings related to their research topic. They can search for recent literature using academic databases, such as PubMed, Google Scholar, or specific journals in the field. | Recent references have been updated using various databases such as PubMed, and Google Scholar among others. | Page 21-23 |
| 2. | Minor grammatical errors: Line 81-85: consider breaking it into two sentences for improved readability. | Lines 81-85 have been broken into two sentences.  All other changes were incorporated after doing a thorough grammar check | Page 4 |
| 3. | Line 88-89: consider revising "until" to "to ensure" for clarity. line 118-122: consider changing "was" to "were" to agree with the plural subject "Antimicrobial susceptibility tests." | The grammar and language correction has been done. | Addressed throughout the revised manuscript |
